# Is the Frequency of Postpartum Breastfeeding Counseling Associated with Exclusive Breastfeeding at Six Months? An Analytical Cross-Sectional Study

**DOI:** 10.3390/children10071141

**Published:** 2023-06-30

**Authors:** Marwah Hassounah, Rufaidah Dabbagh, Afnan Younis

**Affiliations:** Family and Community Medicine Department, College of Medicine, King Saud University, Riyadh 11362, Saudi Arabia; rdabbagh@ksu.edu.sa (R.D.); afnan.younis@gmail.com (A.Y.)

**Keywords:** breastfeeding, counseling, frequency, postpartum, education

## Abstract

Breastfeeding counseling is an essential public health tool in postpartum maternal and infant health. In this study, we aimed to explore the relationship between the frequency of postpartum breastfeeding counseling and the type of feeding outcome at six months. The study design was an analytical, cross-sectional study on mothers of 6–24-month-old children living in Riyadh, Saudi Arabia. We conducted an analysis with descriptive statistics as well as logistic regression models. The findings from our study can be summarized in the following points: First, only 31.9% of the women in our study received postnatal breastfeeding counseling in the first six months after delivery, with the majority receiving fewer than four sessions. Second, there seemed to be a drop in exclusive breastfeeding with time: from 35.3% in the first two months to 29.7% in the second two months and then 20.7% in the final two months. Third, previous exclusive breastfeeding increased the odds of exclusive breastfeeding in the proceeding delivery. Finally, exposure to one or more postnatal breastfeeding counseling sessions increased the odds of exclusive or predominant breastfeeding in the first six months. This study helps to guide decision makers in planning maternal child health services and relevant community-based efforts.

## 1. Introduction/Background

The World Health Organization (WHO) recommends exclusive breastfeeding (human milk, oral rehydration solution, vitamins, and medicines only) for the first six months of life [1]. The percentage of exclusive breastfeeding at six months of age in Saudi Arabia varies in the literature from 2.6% to 37% [2,3]. This is vastly below the WHO 2030 target for each member state to reach a prevalence of 70% exclusive breastfeeding for those under 6 months of age [4]. The literature shows that Saudi Arabia has high breastfeeding initiation rates at delivery, followed by a downward trend after the baby’s first month [2,5,6,7,8,9,10]. There are multiple intrapersonal, interpersonal, environmental, and societal reasons for this repeated observation, with the misconception of not having enough milk being the first reason stated by mothers [2,8,9,10,11,12].

Breastfeeding counseling is a two-way interaction between a counselor and a mother to support breastfeeding practice and help overcome challenges [13]. Counseling employs techniques such as education, practical support of skills, and problem solving [13]. Mothers need counseling to address different levels of knowledge, attitude, and practice. They also need it for professional medical advice in situations such as mastitis and feeding a premature infant, as well as psychological support. Previous studies in Saudi Arabia have focused only on the one-way health education aspect of breastfeeding counseling [8,11,12,14,15]. 

Breastfeeding counseling is a well-documented public health intervention. Globally, the WHO released the first guidelines on the counseling of women to improve breastfeeding practices in 2018. It details the recommended types and frequency of breastfeeding counseling from pregnancy to 24 months (about 2 years) postpartum [16]. However, these recommendations are based on low-quality evidence [16], which emphasizes the role of this study in strengthening the body of knowledge and its translation into action. The WHO’s Baby-Friendly Hospital Initiative (BFHI), first launched in 1991, defines ten steps a hospital should follow to attain a “baby-friendly” status. These include step 10 “Foster the establishment of breastfeeding support groups and refer mothers to them on discharge from the hospital or clinic” [17]. As for the status and quality of BFHI facilities in Saudi Arabia, the available publications are scarce and outdated [3,5]. Recent studies have reported on one of the ten steps, breastfeeding initiation within the first hour after birth, with percentages ranging from 24% to 85% of the women studied [11,18,19].

The literature highlights predictors of positive breastfeeding outcomes in terms of initiation, exclusivity, and duration. The first includes maternal demographic characteristics such as age, where more years predict more positive outcomes [19]. The same applies for having a university education and being a housewife [18,20]. The second includes obstetrics and breastfeeding history including multiparity, vaginal delivery, full-term delivery, the weight of the infant at childbirth, having received counseling after delivery at the facility, a history of breastfeeding their previous child, and colostrum feeding [11,15,18,19,20]. The third includes behavior change constructs such as having high self-efficacy, having intention, and having breastfeeding knowledge [11,15].

In Saudi Arabia, the proportion of women receiving post-delivery breastfeeding counseling on positioning and latching is 60%, whereas 39% are taught how to express milk [21]. The Saudi Public Health Authority’s 2023 National Guideline for Periodic Health Examination recommends (grade B evidence) to start offering breastfeeding counseling at the age of 18 years old when relevant [22]. Furthermore, the Saudi Ministry of Health’s (MOH) maternal health passport reminds physicians to counsel for breastfeeding as early as the first antenatal visit and with every well-baby visit thereafter [23].

Saudi Arabia is undergoing a massive healthcare transformation, with a renewed emphasis on maternal and child health [24]. The transformation’s new paradigm is health promotion and community activation [24]. Understanding the optimal combination of the types and frequency of breastfeeding counseling will aid decision-makers in planning future national maternal health services. The new healthcare transformation is shifting to accountable care organizations (as opposed to the current universal health coverage provided by the government), which serve defined clusters of the population and are paid for by governmental insurance schemes [25]. This shift to privatization raises discussion around the balance between evidence-based practice and cost. Our study adds to the body of knowledge used in evidence-based public health decision making.

The aims of our current study were three-fold. First, we assessed the proportion of four feeding types (exclusive breastfeeding, predominant breastfeeding, mixed breastfeeding, and formula-only feeding) and their distribution across the different participant characteristics. Second, we examined the features of breastfeeding regarding the mother’s previous child and the features of postnatal breastfeeding counseling for the current birth. Third, we assessed the association between frequency of postnatal breastfeeding counseling and self-efficacy with the four feeding types. We hypothesized that exposure to more than six postnatal counseling sessions and having self-efficacy would both increase the odds for exclusive breastfeeding or exclusive and predominant breastfeeding combined [16].

## 2. Methods

### 2.1. Inclusion and Exclusion Criteria

This is an analytical cross-sectional study on women living in Riyadh who have a child between the ages of 6 and 24 months. The study duration was nine months from November 2022 to May 2023. We obtained the King Saud University College of Medicine’s institutional review board approval, E-22-7152, and any necessary administrative approvals from King Khalid University Hospital. The participants provided informed consent before completing the survey. Furthermore, all data was handled with strict confidentiality.

We used convenience sampling to recruit women from King Saud University Medical City and the Snapchat social media platform [26]. The eligibility criteria included any woman living in Riyadh during the study period who had a child between the ages of 6 and 24 months and could read and write in Arabic. The participant was excluded if the 6–24-month-old child was one of multiples (twins, triplets, etc.), born premature, suffered from intrauterine growth restriction, required neonatal intensive care unit admission, or suffered from metabolic syndromes or major health issues requiring a prescribed alteration to feeding practices. In addition, the participants were excluded if the mother had a medical contraindication and/or restriction on breastfeeding such as undergoing chemo- or radio-therapy; suffering from HIV, active herpes infection on the nipple, or uncontrolled epilepsy; or having undergone breast plastic surgery. A total of 73 women were excluded from participation eligibility.

### 2.2. Recruitment and Sample Size

Subject recruitment took place in the waiting areas of the pediatric and family medicine clinics. In addition, a proportion of the participants were recruited through phone numbers obtained from their medical records. A new handle name was created on Snapchat for this research purpose, and we used paid advertisements to be promoted daily to women living in Riyadh over a course of five days. The advertisement had a link that directly took the participants to our online survey. An aspect that is noteworthy is that Snapchat was one of the top used social media platforms in the KSA in 2022, with 24.14 million users (68.8% of Internet users aged 16–64) [27]. Assuming that the population of women of childbearing age was 1,925,600 in Riyadh [28], with a proportion of breastfeeding of 30% and a confidence level of 95%, the estimated sample size was 323 (calculated by the Epi info tool; http://www.openepi.com/SampleSize/SSPropor.htm (accessed on 24 May 2023)).

### 2.3. Data Collection Tool

The data collection tool was an Arabic-language, self-administered, digital Google survey consisting of four sections. The first section asked about social and demographic characteristics, while the second section asked about obstetrics, antenatal, perinatal, and postnatal history. These two sections were constructed by the investigators based on a literature review and the ten steps of the WHO’s Baby Friendly Hospital Initiative (BFHI) [6,8,9,10,11,14,15,29,30]. The third section asked about the exposure to counseling types and frequency (the exposure of interest), which is based on the WHO’s 2018 guidelines on breastfeeding counseling [16] and McFadden et al.’s systemic review and meta-analysis [13]. The fourth section contained three questions on feeding practices (the outcome of interest) and was based on the WHO’s 2018 indicators for assessing infant and young children feeding practices [31]. One indicator, predominant breastfeeding, was used from the WHO’s 2008 version of the same indicators for its cultural relevance, as its definition includes ritual fluids [32]. The outcome feeding types are defined as follows: (1) exclusive breastfeeding is the consumption of human milk, oral rehydration solution, vitamins, and medicines; (2) predominant breastfeeding is where the consumption of human milk is the predominant source of nourishment in addition to liquids such as water and juice, oral rehydration solution, vitamins, medicines, and ritual fluids, e.g., anise tea, sugar water, and soaked dates; (3) mixed feeding combines human milk and formula/and or non-human milk+ food-based fluids; (4) formula feeding only is where formula is the only source of nourishment [31,32]. We asked the same question on feeding for the first two months of the child’s life, the second two months, and the third two months, making up the total of the first six months of the child’s life. This breakdown is recommended by the WHO to accommodate normal feeding variation throughout infancy [31].

The survey was revised for content validity by four experts including an international-board-certified lactation consultant who was also a professor and consultant of family medicine, two family medicine consultants who were certified breastfeeding counselors, and a women’s health public health expert. They were provided with the survey questions and asked whether each was (1) essential, (2) useful but not necessary, or (3) not necessary. The resulting content validity index (CVI) was 0.72. This CVI value is high, which indicates that the test likely measures the construct of interest [33]. Items with content validity ratios (CVR = 0), such as nationality, child sex, father education, and father occupation, were removed to improve the overall content validity of the test. However, 10 items with a CVR of 0.5 were kept in the survey. Furthermore, the experts were given the opportunity to give open-ended comments on each question and the survey in general. Collectively, their input guided further refinement of the survey. Face validity was conducted in the pilot of this survey on 20 mothers after Institutional Review Board approval. The questions were well understood, and the estimated time to complete the survey was noted. Minor changes to the introduction page were made to help with guidance when filling in the data collector’s identifying number.

### 2.4. Data Analysis

We calculated frequencies and percentages for categorical variables, while means and standard deviations (SDs) were calculated for continuous variables. To assess postnatal counseling and self-efficacy’s prediction of the feeding type, we conducted three logistic regression analysis models using different cut-off outcomes (exclusive vs. other types, exclusive or predominant vs. mixed or formula, and any breastfeeding vs. formula only). The outcome feeding type used in the logistic regression was that for the third two months (5 and 6 months of age). For these, we reported adjusted odds ratios and 95% confidence intervals (CIs). In these models, we controlled for maternal age, employment status, monthly household income, previous feeding type, type of delivery (spontaneous vaginal vs. cesarean), skin-to-skin contact at birth, self-efficacy, and breast-feeding counseling frequency. These covariates were controlled for in the models based on their confounding properties in the prediction of breastfeeding type [6,8,9,10,11,14,15,29,30]. All analyses were conducted using the IBM Statistical Package for Social Sciences (SPSS) version 26 (Armonk, NY, USA).

## 3. Results

### 3.1. Participant Characteristics

A total of 323 women responded to the study survey. The mean age of the women was 32 years (SD = 5.22) and almost all were married (98.5%), except for four divorced women and one widow. Most respondents had a college degree or higher (75.9%), were homemakers (65.3%), had an income of less than SAR 10,000 per month, and had a vaginal delivery (69.3%) (Table 1). Furthermore, most of the women had the intention while they were pregnant to breastfeed (94.7%) and had high self-efficacy (76.2%). (Table 1).

### 3.2. Patterns of Feeding the Previous Child

A total of 212 women (65.6%) reported having a previous child. Among these, the majority reported mixed feeding (39.2%). When stratifying these patterns by the current breastfeeding type, it appeared that most of the women reporting current exclusive breastfeeding were also exclusive breast-feeders for their previous child (65.1%). Similarly, most of the formula-only feeders were also previously formula-only feeders (47.4%). This observation was consistent with predominant and mixed feeding too (Table 2). The highest proportion of satisfaction was reported among exclusive breastfeeding women (72.1%), while the lowest was reported among women who only formula fed (35.5%), suggesting that formula-only feeders may have feelings of guilt toward their feeding practices.

### 3.3. Experience with Delivery and Baby-Friendly Hospital-Initiative Practices

Around half of the participants reported delivering at a governmental hospital (59.1%). With respect to assessing the BFHI practices recommended by the WHO [30], the participants’ experiences seem to reflect a care gap. This was most evident for the recommendations of skin-to-skin contact immediately after birth; educating the mother about the risks of bottles, teats, and pacifiers; and receiving post-discharge support (Table 3). Overall, only 41.2% of the women reported experiencing skin-to-skin contact with their baby right after birth, but the highest proportion was reported among women of the predominant breastfeeding group (56.1%). Additionally, only 20.4% recalled being educated about the risks of teats, bottles, and pacifiers, while only 23.2% received post-discharge support.

### 3.4. Features of Postnatal Breastfeeding Counseling at 6 Months Postpartum

**Figure 1:**
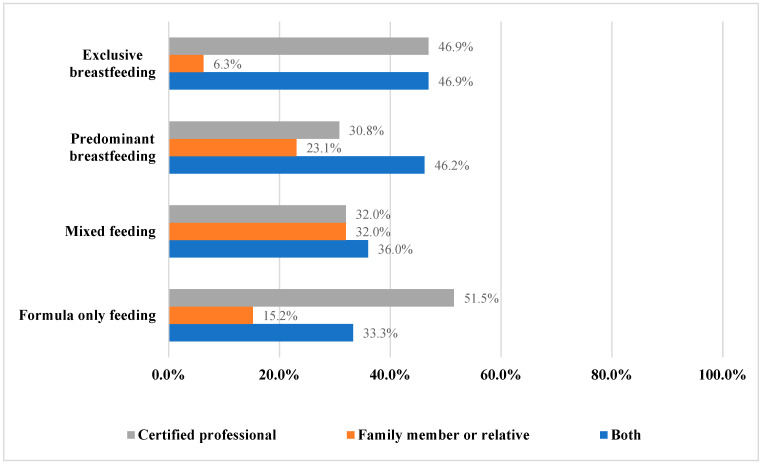
Counseling providers among the women who received breastfeeding counseling (*n* = 103).

Thirty-one percent of the women received postnatal breastfeeding counseling, with the highest proportion reported among women who were exclusive breast-feeders. Among the women who did receive breastfeeding counseling, the majority had fewer than four sessions (Table 3). This feature was consistent in all four feeding groups. Among the 103 women who reported receiving counseling, most of the counseling was provided by a certified professional (Figure 1).

### 3.5. Prediction of Feeding Types

**Figure 2:**
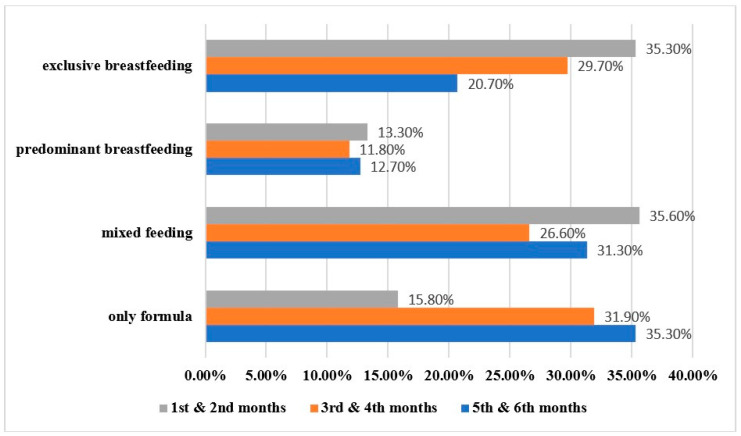
Feeding type prevalence categorized by the first six months postpartum (*n* = 323).

Starting with the feeding type for the first six months, the highest percentages of exclusive breastfeeding and mixed feeding were in the first two months: 114 women (35.3%) and 115 women (35.6%), respectively. By the fifth and sixth month, exclusive breastfeeding declined to 67 women (20.7%) (Figure 2).

The majority of the participants had an income below 20,000 or did not disclose their income. Thus, for the regression analysis, we collapsed the income levels to three groups. Previous feeding type seemed to be an important predictor for the current feeding type. The odds for being an exclusive breast-feeder among those who exclusively breastfed their previous baby was 12.19 times that of the women who were previous formula feeders (AOR = 12.19; 95% CI = 4.98, 29.84). Similarly, previous exclusive breastfeeding was associated with increased odds for exclusive or predominant breastfeeding or any breastfeeding (Table 4). Compared to previous formula feeding, previous predominant breastfeeding was associated with around sixfold odds for exclusive or predominant breastfeeding (AOR = 5.59; 95% CI = 2.09, 14.94) or any breastfeeding (AOR = 5.83; 95% CI = 1.61, 21.19). However, this significant association was not observed when predicting exclusive breastfeeding alone (AOR = 1.21; 95% CI = 0.354, 4.26).

Albeit not statistically significant, high self-efficacy was associated with increased odds for exclusive breastfeeding alone (AOR = 2.13; 95% CI = 0.83, 5.47) and exclusive or predominant breastfeeding (AOR = 1.44; 95% CI = 0.71, 2.93). As anticipated, the odds for exclusive or predominant breastfeeding were significantly greater among the women who had at least one postnatal counseling session compared to the women with no counseling sessions at all (Table 4). However, this significant association was not observed for the prediction of breastfeeding by attending more than six postnatal sessions (AOR = 2.23, 95% CI = 0.47, 10.55).

## 4. Discussion

This study assessed the relationship between breastfeeding counseling frequency and feeding type for the first six months of life. The findings from our study can be summarized in the following points. First, only 31.9% of the women in our study received postnatal breastfeeding counseling in the first six months after delivery, with the majority receiving fewer than four sessions. Second, there seemed to be a drop in exclusive breastfeeding with time: from 35.3% in the first two months to 29.7% in the second two months and then 20.7% in the final two months. Third, previous exclusive breastfeeding increased the odds of exclusive breastfeeding in the proceeding delivery. Finally, exposure to one or more postnatal breastfeeding counseling sessions increased the odds for exclusive or predominant breastfeeding in the first six months.

We were surprised to observe that only around one third of the women received breastfeeding counseling, despite 75% of the women in the study being highly educated and given that they resided in the country’s most resourceful city with versatile healthcare services. The Saudi Ministry of Health established maternal health passports to guide physicians and/or midwives to have two postpartum contact points with mothers. The first is usually scheduled after one week of delivery, while the second visit is between the fourth and sixth weeks postpartum [23]. This facilitates proactive perinatal care in the community. The passport provides forms that include counseling on breastfeeding in the antenatal and postnatal periods. Concurrently, breastfeeding is included in the Ministry of Health well-baby clinic child health passport for mothers to be counselled during visits in the first six months [34]. This collaborative approach yields a total of five post-hospital-discharge breastfeeding counseling intercepts. However, a qualitative study, although not generalizable, revealed that mothers in Al-Ahsa city do not experience this ideal approach lined out by the ministry [35]. Other studies in Saudi Arabia highlighted that postpartum breastfeeding knowledge was obtained from social media and online sources more than health care providers in postpartum health visits [11,15,19]. Furthermore, the 2023 Saudi Clinical Preventive Guidelines give the recommendation of breastfeeding counseling for 18–59-year-old females a grade B rating, but the frequency of counseling is left to the doctor’s clinical judgment to decide [22]. Therefore, our study suggests that this service is not being utilized properly by women in the Saudi community.

There is no established baseline for the percentage of women that receive antenatal or postnatal breastfeeding counseling or even education, whether in community- or hospital-based settings, in Saudi Arabia. We found one published study on community-based breastfeeding counseling activities in Saudi Arabia that described the outcomes of a social media awareness campaign on Twitter [36]. However, one-way education is only one component of counseling. A few local studies on hospital-based breastfeeding counseling efforts have addressed this topic [12,37,38,39]. In Al-Ahsa City Maternal and Child Hospital, a study in 2019 showed that only 47.5% of mothers who delivered there received breastfeeding awareness [37]. That study does not mention what the content of this awareness is and only describes the channels of delivery to be verbal, audio, and print pamphlets [37]. Two hospital-based quasi-experimental studies described the effects of antenatal breastfeeding education videos on the intention to breastfeed [38] and antenatal lactation practical counseling sessions on exclusive breastfeeding practices [39].

In our study, most of the women receiving breastfeeding counseling received fewer than four sessions. Globally, the WHO recommends a minimum of six counseling sessions conducted before and after birth [16]. In a 2019 meta-analysis, being exposed to postpartum counseling four or more times had a greater effect on exclusive breastfeeding in the early postpartum phase when compared to less than four sessions, measured by a 31% (RR 0.69, CI 0.58, 0.82) reduction in the risk of women stopping breastfeeding [13]. For all six months postpartum, we observed this dose–response effect of the number of counseling sessions (up to six sessions) on the feeding types in the outcome results. However, for those who took part in more than six sessions, there seemed to be no added advantage. This finding is of importance to current decision making regarding new national health insurance schemes being developed under the Saudi healthcare transformation. Furthermore, in our prediction of the effect of BFHI practices on feeding outcomes, there was no effect. This could be because our logistic-regression-dependent variable was specified to the feeding outcome in the third set of two months (the fifth and sixth months), and the literature demonstrates that BFHI practices impact the early postpartum period (the first few weeks) rather than later months, which demonstrates the need for community-based support [40,41,42].

The pattern of a decrease in exclusive breastfeeding from the early months postpartum onward is consistent with the literature on Saudi mothers [2,5,6,7,8,9,10]. The reasons for this downward trend in Saudi Arabia are frequently reported to be the wide misconception of low milk supply, going back to work after maternity leave, and maternal health condition [43,44,45]. Similarly, this trend is observed in other high-income countries of the gulf region (the United Arab Emirates initiation prevalence of 95.6% versus exclusive breastfeeding for those under six months of 44.3% [46], Qatar: 33.5% versus 29.3%, Kuwait: 49.9% versus 7.8%, and Oman: 82% versus 23.2%) [47]. The United States’ Centers for Disease Control and Prevention reported a similar trend in 2019, with the initiation of exclusive breastfeeding being at 83.2% and then 24.9% at six months [48]. The low percentage of predominant breastfeeding is interesting, even though we were keen to add it due to its cultural relevance, as seen in clinical practice. This could be explained by our sample having a highly educated majority, so they might be more inclined to follow scientific evidence and are aware of the lack of or discouraging evidence when it comes to cultural and ritual drinks. This is supported by the proportion of those predominantly breastfeeding among those with less than a college education (20.5% row percentage) versus those with a higher level of education (10.2%). Another explanation could be that the respondents did not understand the stated definition of predominant breastfeeding in the survey in comparison to exclusive breastfeeding.

Saudi Arabia is part of the WHO’s Eastern Mediterranean region and classified by the World Bank as a high-income country [49]. The WHO Eastern Mediterranean region has the lowest rates of exclusive breastfeeding under the age of six months among all six regions [50,51]. Globally, low- and middle-income countries have higher exclusive breastfeeding rates for the first six months (top countries: Sri Lanka 80.93%, Rwanda 80.9%, and Burundi 71.9%) as opposed to high-income and high–middle-income countries (top countries: Chile 57%, Palau 52%, and the Netherlands 39%) [52]. First, Chile supports mothers to establish and continue breastfeeding by providing paid maternity leave for more than 26 weeks (Saudi Arabia provides 10 weeks) [53]. Public maternal health services are provided by midwives to uncomplicated pregnant women over seven 20 min antenatal visits. Chilean mothers also gain access to a national oral health program during pregnancy. After mothers give birth, midwives conduct the first two well-baby assessments in which the mothers are screened for postpartum depression. Then, at the time of starting complementary feeds, an appointment with a nutritionist is conducted at five months [54]. Second, Palau is a group of islands in the Western Pacific where primary maternal child health services are funded by the United States [55]. The MOH and non-government entities co-created a community breastfeeding working group that provides support groups, home-based counseling, and text-based breastfeeding support [56]. Since 2006, Palau’s MOH has also taken on the BFHI policy [56]. Last, the Netherlands, also known as Holland, has public maternal health services that rely heavily on midwives and midwife assistants for the care of uncomplicated pregnant women. Midwife assistants provide home services in the antenatal and postnatal periods [57].

This study explores breastfeeding counseling in its interactive definition, and not merely through one-way education. It also has the advantage of describing feeding type by month for the first six months, as recommended by the WHO for this area of research. However, it is not free of limitations. First, a study design that collects postpartum information retrospectively subjects the data to recall bias. We tried to limit the period of recall by defining our target population to mothers of children who are two years old or younger. Second, the sample size was relatively small in comparison to the variables controlled for the multivariate analyses. This resulted in sparse cell counts on stratification, which affected the precision, as reflected in the wide confidence intervals. Furthermore, although we controlled for a list of covariates, we did not control for unmeasured confounders such as chronic conditions or medication use. Thus, we cannot rule out residual confounding effects caused by these unmeasured factors. Finally, due to the convenient sampling strategy, selection bias may have been introduced. Moreover, our sample may not be generalized to the entire Saudi mother population. However, despite these limitations, we believe that our study has provided insights that were previously lacking about breastfeeding behaviors and trends in the local community.

In conclusion, the frequency of postpartum breastfeeding counseling predicted exclusive breastfeeding at six months with a dose–response relationship up to six sessions. Only 31.9% of our studied sample received postnatal breastfeeding counseling, with a majority receiving less than four sessions. Clinical guidelines for postpartum care and health insurance schemes could use this evidence to determine cost-efficient plans.

## Figures and Tables

**Table 1:** Characteristics of the participants by breastfeeding type at six months postpartum.

Characteristics	Exclusive Breastfeeding	Predominant Breastfeeding	Mixed Breastfeeding	Formula-Only Feeding	Total
	*n*= 67	*n* = 41	*n* = 101	*n* = 114	*n* = 323
	Count (%)	Count (%)	Count (%)	Count (%)	Count (%)
Mother’s age, mean (SD)	30.9 (4.25)	33.3 (5.32)	32.2 (5.44)	31.9 (5.44)	32.0 (5.22)
Infant’s age in months, mean (SD)	28.9 (120.5)	15.2 (6.18)	25.1 (98.1)	14.0 (5.96)	20.7 (77.57)
Education level					
Less than college	11 (16.4%)	16 (39.0%)	19 (18.8%)	32 (28.1%)	78 (24.1%)
College or more	56 (83.6%)	25 (61.0%)	82 (81.2%)	82 (71.9%)	245 (75.9%)
Employment status					
Homemaker	50 (74.6%)	28 (68.3%)	64 (63.4%)	69 (60.5%)	211 (65.3%)
Employed	14 (20.9%)	12 (29.3%)	32 (31.7%)	41 (36.0%)	99 (30.7%)
Student	3 (4.5%)	1 (2.4%)	5 (5.0%)	4 (3.5%)	13 (4.0%)
Monthly household income (SAR)					
<10,000	23 (34.3%)	20 (48.8%)	27 (26.7%)	44 (36.6%)	114 (35.3%)
10,000–19,999	24 (35.8%)	9 (22.0%)	36 (35.6%)	26 (22.8%)	95 (29.4%)
20,000–29,999	5 (7.5%)	4 (9.8%)	9 (8.9%)	16 (14.0%)	34 (10.5%)
30,000–39,999	2 (3.0%)	1 (2.4%)	5 (5.0%)	6 (5.3%)	14 (4.3%)
40,000 or more	2 (3.0%)	3 (7.3%)	5 (5.0%)	6 (5.3%)	16 (5.0%)
Did not disclose	11 (16.4%)	4 (9.8%)	19 (18.8%)	16 (14.0%)	50 (15.5%)
Number of live births, mean (SD)	2.3 (1.26)	2.9 (1.53)	2.4 (1.51)	2.5 (1.43)	2.5 (1.44)
Intention to breastfeed during pregnancy					
Yes	64 (95.5%)	39 (95.1%)	98 (97.0%)	105 (92.1%)	306 (94.7%)
No	3 (4.5%)	2 (4.9%)	3 (3.0%)	9 (7.9%)	17 (5.3%)
Self-efficacy: confidence in her own breastfeeding knowledge and skills during pregnancy					
High	60 (86.6%)	33 (80.5%)	73 (72.3%)	80 (70.2%)	246 (76.2%)
Low	7 (10.4%)	8 (19.5%)	28 (27.7%)	34 (29.8%)	77 (23.8%)
Husband’s support for breastfeeding					
Yes	67 (100%)	39 (95.1%)	99 (98.0%)	112 (98.2%)	317 (98.1%)
No	0 (0.0%)	2 (4.9%)	2 (2.0%)	2 (1.8%)	6 (1.9%)
Type of delivery for current birth					
Vaginal delivery	50 (74.6%)	35 (85.4%)	65 (64.4%)	74 (64.9%)	224 (69.3%)
Cesarean	17 (25.4%)	6 (14.6%)	36 (35.6%)	40 (35.1%)	99 (30.7%)

**Table 2:** Patterns of feeding the previous child by breastfeeding type at six months postpartum.

Feature	Exclusive Breastfeeding	Predominant Breastfeeding	Mixed Breastfeeding	Formula-Only Feeding	Total
	*n* = 43.	*n* = 29	*n* = 64	*n* = 76	*n* = 212
	Count (%)	Count (%)	Count (%)	Count (%)	Count (%)
Age of previous child, mean * (SD)	4.8 (2.24)	5.9 (2.79)	5.4 (2.48)	5.5 (2.76)	5.4 (2.58)
Type of feeding for previous child					
Exclusive	28 (65.1%)	6 (20.7%)	7 (10.9%)	5 (6.6%)	46 (21.7%)
Predominant	4 (9.3%)	12 (41.4%)	6 (9.4%)	3 (3.9%)	25 (11.8%)
Mixed	8 (18.6%)	7 (24.1%)	36 (56.3%)	32 (42.1%)	83 (39.2%)
Formula only	3 (7.0%)	4 (13.8%)	15 (23.4%)	36 (47.4%)	58 (27.4%)
Duration of breastfeeding of previous child, mean (SD) **	13.4 (8.45)	13.3 (9.89)	22.77 (124.2)	NA	
Satisfaction with breastfeeding of previous child					
Satisfied	31 (72.1%)	19 (65.5%)	42 (65.6%)	27 (35.5%)	119 (56.1%)
Unsatisfied	11 (25.6%)	8 (27.6%)	17 (26.6%)	40 (52.6%)	76 (35.8%)
Don’t know	1 (2.3%)	2 (6.9%)	5 (7.8%)	9 (11.8%)	17 (8.0%)

* Age in years; ** any type of breastfeeding: exclusive, predominant, or mixed, duration in months. NA not applicable.

**Table 3:** Features of postnatal breastfeeding counseling by breastfeeding type, six months postpartum.

Postnatal Counseling Feature	Exclusive Breastfeeding	Predominant Breastfeeding	Mixed Breastfeeding	Formula-Only Feeding	Total
	*n* = 67	*n* = 41	*n* = 101	*n* = 114	*n* = 323
	Count (%)	Count (%)	Count (%)	Count (%)	Count (%)
Place of birth					
Governmental hospital	36 (53.7%)	33 (80.5%)	59 (58.4%)	63 (55.3%)	191 (59.1%)
Private hospital	31 (46.3%)	8 (19.5%)	42 (41.6%)	51 (44.7%)	132 (40.9%)
Skin-to-skin contact at birth					
Yes	28 (41.8%)	23 (56.1%)	39 (38.6%)	43 (37.7%)	133 (41.2%)
No	39 (58.2%)	18 (43.9%)	62 (61.4%)	71 (62.3%)	190 (58.8%)
Breastfeeding initiation within first hour					
Yes	26 (38.8%)	22 (53.7%)	39 (38.6%)	39 (34.2%)	126 (39.0%)
No	41 (61.2%)	19 (46.3%)	62 (61.4%)	75 (65.8%)	197 (61.0%)
Practiced rooming-in					
Yes	54 (67.2%)	28 (68.3%)	62 (61.4%)	52 (45.6%)	187 (57.9%)
No	22 (32.8%)	13 (31.7%)	39 (38.6%)	62 (54.4%)	136 (42.1%)
Encouraged to practice responsive feeding and to know feeding cues					
Yes	22 (32.8%)	12 (29.3%)	42 (41.6%)	39 (34.2%)	115 (35.6%)
No	45 (67.2%)	29 (70.7%)	59 (58.4%)	75 (65.8%)	208 (64.4%)
Hospital encouraged or provided bottles, teats, and/or pacifiers					
Yes	10 (14.9%)	9 (22.0%)	20 (19.8%)	27 (23.7%)	66 (20.4%)
No	57 (85.1%)	32 (78.0%)	81 (80.2%)	87 (76.3%)	257 (79.6%)
Hospital arranged for post-discharge breastfeeding support (ex: chat group for inquiries)					
Yes	20 (29.9%)	4 (9.8%)	20 (19.8%)	31 (27.2%)	75 (23.2%)
No	47 (70.1%)	37 (90.2%)	81 (80.2%)	83 (72.8%)	248 (76.8%)
Received antenatal breastfeeding education					
Yes	29 (43.3%)	14 (34.1%)	46 (45.5%)	49 (43.0%)	138 (42.7%)
No	38 (56.7%)	27 (65.9%)	55 (54.5%)	65 (57.0%)	185 (57.3%)
At the hospital, after delivery, received postnatal breastfeeding counseling					
Yes	40 (59.7%)	25 (61.0%)	68 (67.3%)	77 (67.5%)	210 (65.0%)
No	27 (40.3%)	16 (39.0%)	33 (32.7%)	37 (32.5%)	113 (35.0%)
Received postnatal breastfeeding counseling during the first six months after delivery					
Yes	32 (47.8%)	13 (31.7%)	25 (24.8%)	33 (28.9%)	103 (31.9%)
No	35 (52.2%)	28 (68.3%)	76 (75.2%)	81 (71.1%)	220 (68.1%)
Type of counseling received *					
Face-to-face	10 (14.9%)	3 (7.3%)	8 (7.9%)	15 (13.2%)	36 (11.1%)
Virtual	13 (19.4%)	4 (9.8%)	9 (8.9%)	10 (8.8%)	36 (11.1%)
Both	8 (11.9%)	4 (9.8%)	6 (5.9%)	7 (6.1%)	25 (7.7%)
None	36 (53.7%)	30 (73.2%)	78 (77.2%)	82 (71.9%)	226 (70.0%)
Frequency of breastfeeding counseling sessions by 6 months postpartum (times)					
None	35 (52.2%)	28 (68.3%)	76 (75.2%)	81 (71.1%)	220 (68.1%)
1–3	20 (29.9%)	9 (22.0%)	21 (20.8%)	24 (21.1%)	74 (22.9%)
4–6	9 (13.4%)	1 (2.4%)	2 (2.0%)	6 (5.3%)	18 (5.6%)
>6	3 (4.5%)	3 (4.5%)	2 (2.0%)	3 (2.6%)	11 (3.4%)

* Face-to-face (e.g., hospital, clinic, or home) or virtual (e.g., telephone, messaging applications such as WhatsApp and Telegram, video call, or social media websites).

**Table 4:** Predicting breastfeeding type at six months postpartum by frequency of exposure to postnatal breastfeeding counseling sessions, self-efficacy, and other covariates.

Participant Characteristic	Exclusive Breastfeeding vs. Other Types	Exclusive or Predominant Breastfeeding vs. Mixed or Formula	Any Breastfeeding (Exclusive, Predominant, or Mixed) vs. Formula
	AOR (95% CI)	AOR (95% CI)	AOR (95% CI)
Maternal age	0.95 (0.88, 1.02)	1.00 (0.94, 1.06)	0.99 (0.94, 1.05)
Employment			
Homemaker	Ref	Ref	Ref
Employed	0.45 (0.20, 1.03)	0.66 (0.34, 1.29)	0.61 (0.34, 1.10)
Student	1.34 (0.29, 6.06)	1.12 (0.29, 4.27)	1.47 (0.41, 5.23)
Education level			
Less than college	Ref	Ref	Ref
College or more	2.19 (0.92, 5.21)	1.01 (0.52, 1.96)	1.53 (0.85, 2.77)
Monthly household income (SAR)			
<10,000	Ref	Ref	Ref
≥10,000	1.28 (0.61, 2.69)	0.79 (0.42, 1.49)	1.26 (0.70, 2.27)
Did not disclose	1.23 (0.47, 3.18)	0.62 (0.27, 1.42)	1.36 (0.64, 2.87)
Previous feeding type			
Formula only	Ref	Ref	Ref
Mixed	0.58 (0.23, 1.49)	0.61 (0.29, 1.27)	1.25 (0.68, 2.30)
Predominant	1.21 (0.35, 4.26)	**5.59 (2.09, 14.94)**	**5.83 (1.61, 21.19)**
Exclusive	**12.19 (4.98, 29.84)**	**9.26 (3.99, 21.48)**	**6.55 (2.35, 18.25)**
Type of delivery for current birth			
Vaginal delivery	0.95 (0.45, 2.00)	1.69 (0.90, 3.18)	1.09 (0.63, 1.88)
Cesarean	Ref	Ref	Ref
Skin-to-skin contact at birth			
Yes	0.98 (0.50, 1.92)	1.29 (0.74, 2.26)	1.19 (0.71, 1.99)
No	Ref	Ref	Ref
Self-efficacy			
High	2.13 (0.83, 5.47)	1.44 (0.71, 2.93)	1.10 (0.62, 1.97)
Low	Ref	Ref	Ref
Postnatal counseling frequency			
None	Ref	Ref	Ref
1–3	**2.99 (1.40, 6.39)**	**2.29 (1.20, 4.24)**	1.34 (0.74, 2.44)
4–6	**7.54 (2.42, 23.52)**	**4.24 (1.21, 12.69)**	1.22 (0.42, 3.59)
>6	2.23 (0.47, 10.55)	**4.03 (1.03, 15.78)**	1.59 (0.37, 6.78)

Bold text notes statistical significance. In these models we controlled maternal age, employment status, monthly household income, previous feeding type, type of delivery (spontaneous vaginal vs. cesarean), skin-to-skin contact at birth, self-efficacy, and breast-feeding counseling frequency.

## Data Availability

Data unavailable.
